# 96. A Candidate Respiratory Syncytial Virus (RSV) Prefusion F Protein Investigational Vaccine (RSVPreF3 OA) Is Immunogenic when Administered in Adults ≥ 60 Years of Age: Results at 6 Months after Vaccination

**DOI:** 10.1093/ofid/ofac492.174

**Published:** 2022-12-15

**Authors:** Tino F Schwarz, Shinn-Jang Hwang, Pedro P Ylisastigui, Chiu-Shong Liu, Kenji Takazawa, Makoto Yono, John E Ervin, Charles Andrews, Charles Fogarty, Tamara Eckermann, Delphine Collete, Magali de Heusch, Nathalie De Schrevel, Bruno Salaun, Marc Lievens, Céline Maréchal, Phoebe Nakanwagi, Veronica Hulstrøm

**Affiliations:** Klinikum Würzburg Mitte, Campus Juliusspital, Würzburg, Germany, Wuerzburg, Bayern, Germany; En Chu Kong Hospital, New Taipei City, Taipei, Taiwan; Alliance For MultiSpecialty Research, Fort Myers, Florida; China Medical University Hospital, Taichung, Taichung, Taiwan; Medical Corporation Shinanokai Shinanozaka Clinic, Shinjuku-ku, Tokyo, Japan; Nishi-Kumamoto Hospital, Souseikai, Kumamoto, Kumamoto, Japan; AMR Kansas City, Kansas City, Missouri; Diagnostics Research Group, San Antonio, Texas; Spartanburg Medical Research, Spartanburg, South Carolina; Hausarztpraxis Heimeranplatz, München, Bayern, Germany; GSK, Rixensart, Brabant Wallon, Belgium; GSK, Rixensart, Brabant Wallon, Belgium; GSK, Rixensart, Belgium, Rixensart, Brabant Wallon, Belgium; GSK, Rixensart, Brabant Wallon, Belgium; GSK, Rixensart, Brabant Wallon, Belgium; GSK, Rixensart, Brabant Wallon, Belgium; GSK, Rixensart, Brabant Wallon, Belgium; GSK, Wavre, Belgium, Wavre, Brabant Wallon, Belgium

## Abstract

**Background:**

RSV infections are frequent and can lead to respiratory complications in older adults (OA). However, there is no licensed RSV vaccine yet. Here we present immunogenicity results up to month (M) 6 after vaccination with the RSVPreF3 OA.

**Methods:**

In this phase 3 multi-country ongoing study (NCT04732871), adults ≥ 60 years of age were randomized (3:1:1) to receive RSVPreF3 OA and to be followed up for 3 years. All participants received a dose of RSVPreF3 on day (D) 1. Humoral immune (HI) and cell-mediated immune (CMI) responses were measured in subsets of participants at pre-vaccination (D1), D31 and M6. HI outcomes included RSV-A and RSV-B neutralizing antibody (NAb) geometric mean titers (GMTs) and RSVPreF3-specific immunoglobulin G (IgG) antibody geometric mean concentrations (GMCs). The CMI response was assessed in terms of frequency of RSVPreF3-specific CD4^+^ T-cells and CD8^+^ T-cells expressing at least 2 activation markers including at least 1 cytokine among CD40L, 4-1BB, IL-2, TNF-α, IFN-γ, IL-13, IL-17 (polypositive T-cells).

**Results:**

A total of 1653 participants received a dose of RSVPreF3 OA. Of these, 987 participants were included in the HI subset and 566 in the CMI subset at D1. The RSV-A and RSV-B GMTs and RSVPreF3-specific IgG GMCs increased between D1 and D31 followed by a decline until M6. At D31, RSV-A and RSV-B NAb GMTs were 10.5-fold and 7.8-fold higher than pre-vaccination (Figure), and RSVPreF3-specific IgG antibody GMCs was 12.2-fold higher than pre-vaccination levels. At M6, the RSV-A and RSV-B GMTs were 4.4-fold and 3.5-fold, and RSVPreF3-specific IgG antibody GMCs were 4.7-fold above pre-vaccination levels. The RSVPreF3-specific polypositive CD4^+^ T-cell median frequency (events/10^6^ cells) increased from 191 (below assay quantification limit) to 1339 at D31 and declined to 666 (above assay quantification limit) by M6. No RSVPreF3-specific CD8^+^ T-cell response was detected after RSVPreF3 OA vaccination.

**Figure:**
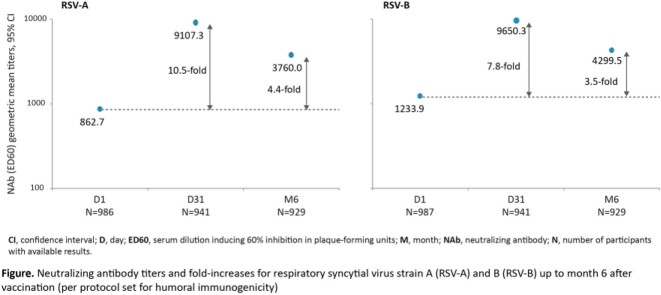

**Conclusion:**

In adults ≥ 60 years of age, 1 dose of RSVPreF3 OA was shown to be immunogenic, with both high HI and specific CMI responses at D31 post-vaccination and remained 3.5–4.7 fold above pre-vaccination levels at M6. This study will continue to monitor the immunogenicity of RSVPreF3 OA up to 3 years.

**Funding:**

GlaxoSmithKline Biologicals SA.

**Disclosures:**

**Tino F. Schwarz, Prof. Dr. MD**, Biogen, Merck-Serono, Pfizer, Alexion, Bavarian Nordic, Janssen-Cilag, AstraZeneca, Biontech, MSD: Grants|GlaxoSmithKline Biologicals SA: Honoraria **John E. Ervin, MD**, The Alliance for Multispecialty Research – KCM: Contractual agreement for conduct of study protocol **Charles Andrews, MD**, GlaxoSmithKline Biologicals SA: Institutional grant|Merck and Boehringer Ingelheim: Consulting fees outside of the submitted work **Delphine Collete, PhD**, GlaxoSmithKline Biologicals SA: Employee|GlaxoSmithKline Biologicals SA: Stocks/Bonds **Magali de Heusch, PhD**, GlaxoSmithKline Biologicals SA: Employee|GlaxoSmithKline Biologicals SA: Stocks/Bonds **Nathalie De Schrevel, PhD**, GlaxoSmithKline Biologicals SA: Employee|GlaxoSmithKline Biologicals SA: Ownership Interest **Bruno Salaun, PhD**, GlaxoSmithKline Biologicals SA: Employee|GlaxoSmithKline Biologicals SA: Stocks/Bonds **Marc Lievens, MSc**, GlaxoSmithKline Biologicals SA: Employee|GlaxoSmithKline Biologicals SA: Stocks/Bonds **Céline Maréchal, PhD**, GlaxoSmithKline Biologicals SA: Employee|GlaxoSmithKline Biologicals SA: Stocks/Bonds **Phoebe Nakanwagi, Master’s in Biostatistics**, GlaxoSmithKline Biologicals SA: Employee **Veronica Hulstrøm, PhD MD**, GlaxoSmithKline Biologicals SA: Employee.

